# Diclofenac Inhibits Tumor Growth in a Murine Model of Pancreatic Cancer by Modulation of VEGF Levels and Arginase Activity

**DOI:** 10.1371/journal.pone.0012715

**Published:** 2010-09-15

**Authors:** Nina Mayorek, Nili Naftali-Shani, Myriam Grunewald

**Affiliations:** Developmental Biology and Cancer Research, Institute for Medical Research Israel-Canada (IMRIC), The Hebrew University-Hadassah Medical School, Jerusalem, Israel; The University of Hong Kong, Hong Kong

## Abstract

**Background:**

Diclofenac is one of the oldest anti-inflammatory drugs in use. In addition to its inhibition of cyclooxygenases (COX), diclofenac potently inhibits phospholipase A_2_ (PLA_2_), thus yielding a broad anti-inflammatory effect. Since inflammation is an important factor in the development of pancreatic tumors we explored the potential of diclofenac to inhibit tumor growth in mice inoculated with PANCO2 cells orthotopically.

**Methodology/Principal Findings:**

We found that diclofenac treatment (30 mg/kg/bw for 11 days) of mice inoculated with PANC02 cells, reduced the tumor weight by 60%, correlating with increased apoptosis of tumor cells. Since this effect was not observed *in vitro* on cultured PANCO2 cells, we theorized that diclofenac beneficial treatment involved other mediators present in *vivo*. Indeed, diclofenac drastically decreased tumor vascularization by downregulating VEGF in the tumor and in abdominal cavity fluid. Furthermore, diclofenac directly inhibited vascular sprouting *ex vivo*. Surprisingly, in contrast to other COX-2 inhibitors, diclofenac increased arginase activity/arginase 1 protein content in tumor stroma cells, peritoneal macrophages and white blood cells by 2.4, 4.8 and 2 fold, respectively. We propose that the subsequent arginine depletion and decrease in NO levels, both in serum and peritoneal cavity, adds to tumor growth inhibition by malnourishment and poor vasculature development.

**Conclusion/Significance:**

In conclusion, diclofenac shows pronounced antitumoral properties in pancreatic cancer model that can contribute to further treatment development. The ability of diclofenac to induce arginase activity in tumor stroma, peritoneal macrophages and white blood cells provides a tool to study a controversial issue of pro-and antitumoral effects of arginine depletion.

## Introduction

Inflammation is highly related to both carcinogenesis and to progression of tumor growth [1]. Epidemiological studies show that chronic inflammation predisposes patients to cancer development and long-term use of non-steroidal anti-inflammatory drugs (NSAIDs) reduces the risk of several cancers [2].

Cyclooxygenases (COX-1 and -2) are rate-limiting enzymes in the production of prostaglandins (PGs) from arachidonic acid.

COX-1 expression is generally constitutive, while COX-2 is usually induced by stimuli involved in inflammatory responses. Prostaglandin E2 (PGE2), a primary metabolite of COX-2, has been shown to promote cell survival, proliferation, and angiogenesis and inhibit apoptosis and antitumor immune response, all processes promoting cancer development [3].

Pancreatic cancer is a lethal disease with very limited options for treatment. Chronic pancreatitis predisposes individuals to developing pancreatic ductal adenocarcinoma [4] and pancreatic tumor stroma is characterized by the variety of inflammatory cells [5] suggesting the involvement of inflammatory processes in this cancer. COX-2 is overexpressed in approximately 75% of human carcinomas including those of the pancreas. Recent studies in transgenic mice [6], [7], [8] have shown that COX-2 overexpression in the exocrine pancreas induced pancreatitis-like-state. The onset of the cancer in these mice was prevented by maintaining mice on selective COX-2 inhibitors, further demonstrating the important role of inflammation on pancreatic cancer development.

Human trials to prevent colon cancer were recently conducted using COX-2 inhibitors. Despite encouraging results in colon cancer prevention and reduction in the incidence of colon adenomas, NSAIDs and selective COX-2 inhibitors can cause serious cardiovascular and gastrointestinal side effects and therefore are not routinely recommended for these diseases [9], [10].

Diclofenac, one of the oldest NSAIDs has been in use since 1976. Diclofenac inhibits cyclooxygenases non-selectively. Studies in humans demonstrated that it reduces 94% of COX-2 and 49% of COX-1 activity [11]. In vitro it potently inhibits phospholipase A2 (PLA2) [12], the enzyme which liberates arachidonic acid and lysophospholipid to generate a family of pro-inflammatory eicosanoids (including PGE2) and platelet activating factor. Additional evidence suggests that diclofenac also inhibits PLA2 in *vivo*. PLA2 is believed to play a key role in the first steps of the inflammatory cascade leading to acute pancreatitis [12]. The initial study [13], followed by several other groups, has shown that diclofenac can significantly decrease the rate of acute pancreatitis caused by endoscopic retrograde cholangiopancreatography.

In light of diclofenac ability to inhibit potently both COX-2 and PLA2 we have explored its anticancer potential in a mouse model of pancreatic cancer. Here we report that diclofenac treatment causes a 60% decrease in tumor size. This is due to increased apoptosis of tumor cells. We provide evidence that this effect is indirect and operates through at least two mechanisms. First, diclofenac yields a significant antiangiogenic effect, as demonstrated by a strong reduction in vascular endothelial growth factor (VEGF) tumor content, decreased microvascular density and morphological changes in tumor blood vessels. Second, diclofenac treatment remarkably increases arginase activity in tumor stroma cells, peritoneal macrophages and white blood cells. This is an unexpected finding since COX-2 inhibitors were shown to reduce tumor growth through arginase inhibition [14], [15] Our results are in line with earlier studies, which point to antitumoral [16] and antiangiogenic effect [17] of arginine depletion and support the rather controversial approach of anticancer treatment by enhancing arginase activity [18], [19].

## Methods

### Ethics statement

Experimental animal procedures were approved by the Ethical Committee of the Hebrew University, which acts under the surveillance of the AAALAC International.

### Animals and PANCO2 cell line

Female CB6F1 mice (9–10 weeks old) and male Sprague-Dawley rats (200 g) were from Harlan, Israel. Female CX3CR1^GFP/+^ mice [20] were generously provided by S. Jung (Rehovot, Israel). In these mice a GFP reporter is driven by the CX3CR1 promoter. Activity of this chemokine receptor promoter is restricted to mononuclear myeloid cells, including all circulating CD116^+^ monocytes, and is essentially absent from cells of the lymphoid lineage. All experiments except the experiment presented in Fig S3 were performed using CB6F1 mice.

PANC02 cells were a generous gift from M Dauer (Munich, Germany). Cells were maintained in RPMI with 10% fetal bovine serum (FBS), 2 mM L-glutamine, 100 U/ml penicillin and 100 µg/ml streptomycin.

### Orthotopic-syngeneic model of pancreatic cancer

Mice were anesthetized with a mixture of ketamine-xylazine. The pancreas was injected with 4×10^5^ (CB6F1 mice) or 6×10^5^ (CX3CR1^GFP/+^ mice) PANC02 cells in 30 µl phosphate buffered saline (PBS). Mice were removed from an experiment if a peritoneal leakage was suspected. Sham operated mice underwent the same procedure and were injected with 30 µl PBS. Mice were divided into groups of 6 to 8 animals per group. The treated group received 30 mg/kg b.w. diclofenac in drinking water starting from day 3 after inoculation. The dosage was calculated based on the estimate that a mouse drinks about 3 ml water per day. Mice were killed 14 days after inoculation unless specified otherwise.

Blood for white blood cells (WBC) preparation, VEGF and nitrogen oxide (NO) concentration was taken from heart. Peritoneal cavity was flushed with 2 ml of PBS with 0.1% bovine serum albumin (BSA) for macrophages preparation, for measurement of VEGF and for NO concentrations.

### Peritoneal cells and peritoneal macrophages isolation

Peritoneal cells were obtained by centrifugation of peritoneal cavity flush. Peritoneal macrophages were isolated from peritoneal cells by differential adhesion to plastic. The macrophages were cultured in RPMI with 10% FBS, 2 mM L-glutamine, 100 U/ml penicillin and 100 µg/ml streptomycin for 20 hrs. To measure arginase activity, cells were washed with PBS and dissolved in 0.1% TritonX100 in water for 30 min at room temperature.

### Tumor homogenates

Tumor homogenates were prepared from tumor samples which were stored at −80°C, in water containing 0.1% Triton X 100 and protease inhibitor cocktail (Sigma P8340). Homogenates were centrifuged for 30 min at 13400 g and the supernatants were used for measuring arginase activity, arginase 1, smooth muscle actin and VEGF content and caspase-3 activation.

### Bone marrow cells isolation

Tibias and femurs of tumor bearing mice were removed and flushed with PBS. After centrifugation of harvested cells on a Ficoll gradient at 4000 g for 20 min at room temperature mononuclear cells were isolated with PE-conjugated primary antibody anti–CD 115 and anti-PE microbeads (Biolegends). CD-115 positive and negative cells were analyzed for arginase activity.

### White blood cells isolation (WBC)

WBC were isolated using Ficoll density gradient (Amersham). WBC fraction was washed, cells were counted, lysed in water containing 0.1% Triton X 100 and analyzed for arginase 1 protein content.

### Aortic ring sprouting assay

Aortic rings were prepared from thoracic aortas of Sprague-Dawley rats, as described [21]. Rings were embedded in collagen gel and maintained in Bio MpM medium (Biological Industries, Israel). The treatment with 10 µM diclofenac started one day after seeding and lasted for 5 days. Rings were then fixed in 4% buffered formalin, stained with 0.02% crystal violet in absolute ethanol. Sprouting area was evaluated using the Image Pro program.

### Caspase-3 activation

Caspase-3 activation was measured in tumor homogenates by separating the non-cleaved and cleaved caspase-3 on a 20% acrylamide SDS-PAGE electrophoresis gel and blotting with monoclonal anti–caspase-3 (Cell Signaling).

### VEGF and NO content

VEGF content was measured in serum, in supernatant of peritoneal cavity flush, in tumor homogenates, PANC02 and cultured macrophages using the mouse VEGF Quantikine immunoassay kit (R &D systems).

NO content was measured in the serum and in the supernatant of peritoneal cavity flush using a nitrate/nitrite colorimetric assay kit (Cayman Chemical Company).

### Arginase activity

Arginase activity was measured as described [22]. 100 µg of protein was assayed for 10 min at 37°C for peritoneal cells and macrophages, 30 min for tumor homogenates and 2 hours for bone marrow cells. Protein content was measured in each sample using the micro BCA protein assay kit (Thermo Scientific).

### Arginase 1 and smooth muscle actin protein content

Proteins from tumor homogenates, WBC lysates and GFP positive cells isolated from tumors were separated on 10% acrylamide SDS-PAGE electrophoresis gel and blotted either with monoclonal arginase-1(BD Transduction Laboratories) or monoclonal smooth muscle actin (Sigma).

### Isolation of mononuclear myeloid cells from tumors

Tumors were extracted from CX3CR1^GFP/+^ mice and digested with collagenase (400 units/ml; Sigma and Dnase 0.33 mg/ml; Sigma) for 45 min at 37°C. GFP positive population was purified by high speed cell sorting using FACS Aria (Becton, Dickinson). Cells were counted and lysed in water containing 0.1% Triton X 100 and analyzed for arginase 1 protein content.

### PANCO2 cells rate of growth

PANC02 cells were seeded in RPMI supplemented with 10% FBS, 2 mM L-glutamine, 100 U/ml penicillin and 100 µg/ml streptomycin. Diclofenac (10 or 50 µM) was added the following day and after 4 days of treatment, cells content was measured using a cell proliferation kit (Biological Industries, Israel).

### Immunohistochemistry, TUNEL staining and morphometry

Paraffin sections of tumors were stained using the following primary antibodies: rat anti-mouse F4/80 (Serotec), monoclonal anti- arginase-1(BD Transduction Laboratories), monoclonal anti- α-smooth muscle actin (Sigma), monoclonal anti- Ki 67 (Neomarkers), rabbit anti- phosphor-histone H3 (Cell Signaling), rabbit anti-VEGF (Calbiochem) and monoclonal anti- von Willebrandt factor (DACO). Horseradish peroxidase (HRP) conjugated secondary antibodies were used for chromogenic detection.

TUNEL staining [23] was performed using in situ cell death detection kit (Roche).

Quantification of immunostaning data was performed either by high power field analysis counting of at least 10 areas/slide of 4 different tumors in each group or with the aid of image analysis system (Ariol SL-50).

### Statistical analysis

All data were analyzed with SigmaStat software (Aspire software international). A Mann-Whitney test was used to calculate significant differences between untreated and diclofenac treated samples. A value of p≤0.05 was accepted as significant.

Sample size and number of repetitions are indicated in legends.

## Results

### Diclofenac inhibits tumor growth in an orthotopic model of pancreatic cancer in mice

**Figure 1:**
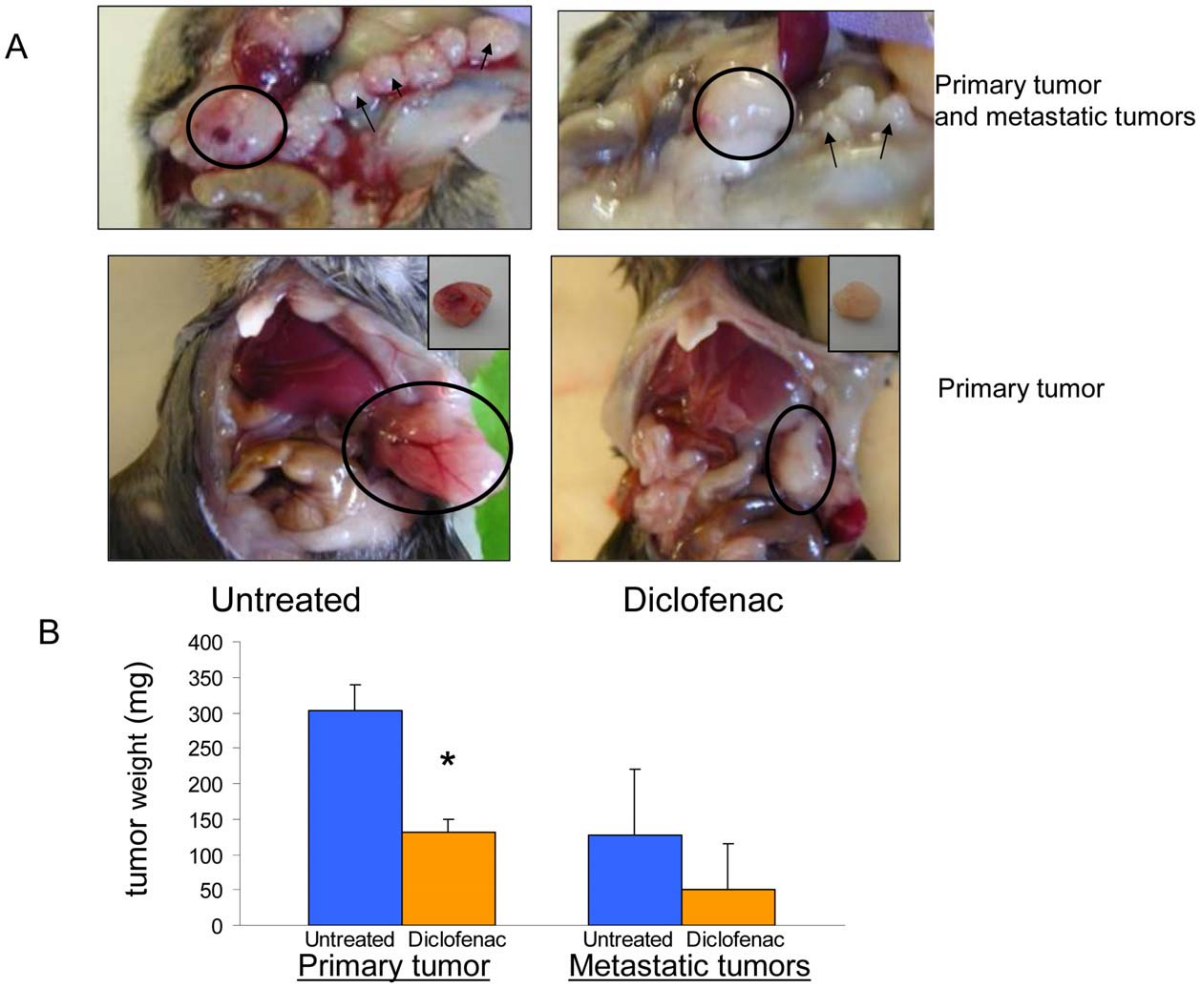
Diclofenac inhibits primary tumors growth in an orthotopic model of pancreatic cancer in mice. Mice were inoculated with 4×10^5^ PANC02 cells into the tail part of pancreas. On day 3 after inoculation (the day of inoculation was designated as day 1) mice were randomized into untreated and diclofenac treated groups (7–8 mice in each group) and diclofenac 30 mg/kg b.w treatment was started. On day 14 after inoculation mice were killed and primary and metastatic tumors were removed and weighed. A, a representative photographs of *in situ* primary (*circles*) and metastatic tumors (*arrows*). Inserts show isolated primary tumors. B, Mean ± SE of tumor weights of 8 untreated and 7 diclofenac treated mice * P≤0.01. The representative experiment out of four is shown.

PANC02 cells were injected into the tail of the pancreas. After 4 days cancer cells formed small tumors of approximately 4 mm length. Tumors developed rapidly; at day 8 they had doubled in length and consistently metastasized to the peritoneal area of the abdominal incision executed for tumor inoculation. At day 14, tumors which grew at the site of the inoculation (primary tumors), weighed 300–500 mg and were highly vascularized. The metastatic tumors at the abdominal cut and in spleen were also prominent (Fig 1A).

Three to four weeks after inoculation, the peritoneal cavity was full of bloody ascites. Numerous metastatic tumors were found in the peritoneal cavity, including lymph nodes of the alimentary track, and mice began to die. Metastatic tumors on liver or lungs were not detected.

Subsequent experiments were therefore terminated at day 14 after inoculation. The mice appeared normal at this stage and there was no loss of body weight.

Diclofenac (30 mg/kg bw) was given daily in drinking water starting at day 3 after inoculation. Its effects were examined after 11 days of treatment. As shown in Fig 1B, the weight of the primary tumor (growing in the pancreas) was significantly lower (60%) in diclofenac treated mice as compared to untreated animals. Metastatic tumors which grew in the abdominal cut also weighed less in diclofenac treated mice, in all experiments, yet this effect did not achieve statistical significance.

### Diclofenac increases apoptosis of tumor cells *in vivo*, but not *in vitro*

The decreased tumor weight observed in diclofenac treated mice led us to examine proliferation and apoptosis of tumor cells.

Tumors stained for Ki 67 (present during all active phases of the cell cycle: G1, S, G2, and mitosis) and of phospho-histone H3 (the specific marker of mitotic cells) showed no difference between untreated and diclofenac treated mice, Fig S1.

**Figure 2:**
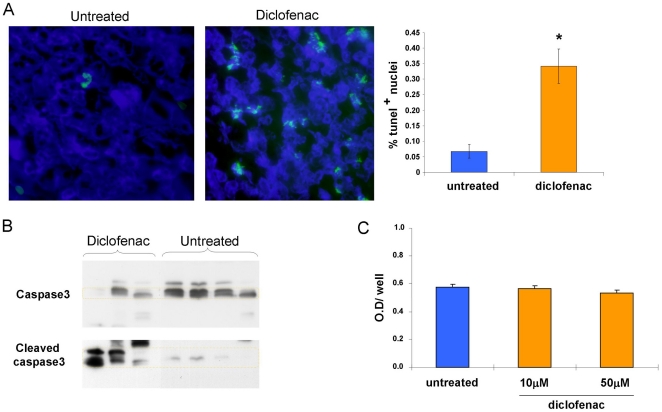
Diclofenac increases apoptosis of tumor cells *in vivo* but not *in vitro*. A and B, mice were inoculated with PANC02 cells and treated with diclofenac as described in Figure 1. A, *lef*t, TUNEL staining superpositioned over DAPI staining of fixed tumors, *right*, mean± SE % of TUNEL positive nuclei out of DAPI stained nuclei. About 9000 nuclei from tumors of 4 untreated and 4 diclofenac treated mice were counted * P≤0.001. B, Caspase-3 activation in tumor homogenates of 4 untreated and 3 diclofenac treated mice was measured as described in Materials and Methods, one out of two experiments. C, PANC02 (3000 cells/well) were seeded in 96-NUNC wells. The day after seeding 10 or 50 µM diclofenac was added and cells were incubated for additional 4 days. The number of cells was measured using a Cell Proliferation Kit. Mean ±SE of OD of 6 wells in each group. One experiment representative of 3.

However, as shown in Fig 2, diclofenac treatment increased apoptosis by 6 fold, as indicated by TUNEL staining of tumors and quantification (Fig 2A). This finding was further supported by an enhanced presence of cleaved caspase 3 in homogenates from tumors excised from diclofenac treated mice (Fig 2B).

The enhanced apoptosis caused by diclofenac *in vivo* could not be recapitulated *in vitro*. PANC02 cells cultured for 4 days in the presence of 10 and 50 µM of diclofenac grew at the same rate as untreated cells (Fig 2C) indicating that the *in vivo* effects of diclofenac require some mediators that are absent in the *in vitro* system.

### Antiangiogenic effect of diclofenac *in vivo* and *ex vivo*

**Figure 3:**
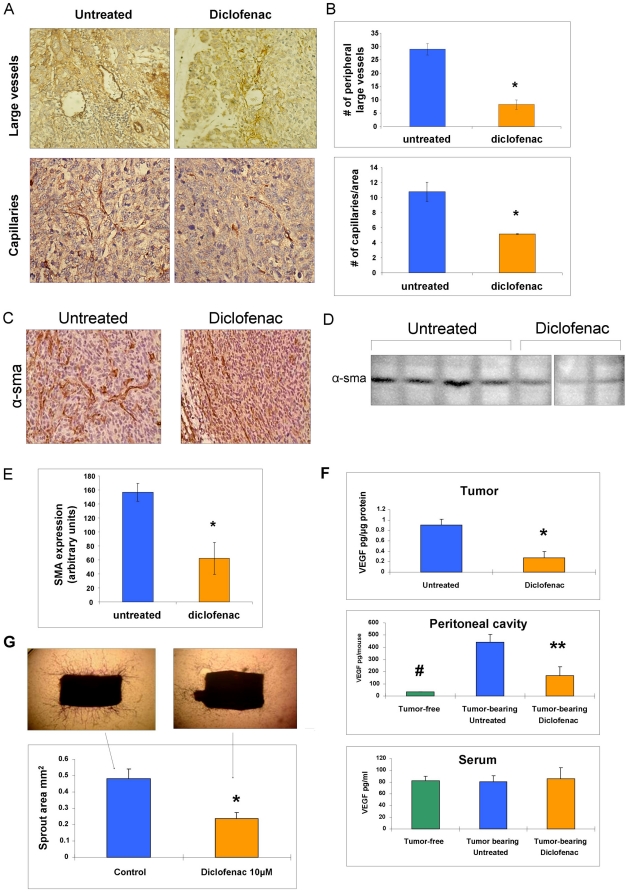
Diclofenac decreases angiogenesis. A–F, mice were inoculated with PANC02 cells and treated with diclofenac as described in Figure 1. A–D, diclofenac treatment causes morphological changes in tumor blood vessels. A,Von Willebrand staining of large peripheral vessels (*top)* and capillaries (*bottom*). B, Mean± SE of large peripheral vessels/slide. counted around the periphery of each slide (*top*) and mean± SE of capillaries/area counted in 10 different central areas of tumors under X40 magnification (*bottom*). Vessels count was evaluated in tumors of 4 untreated and 4 diclofenac treated mice, * P≤0.01. C, smooth muscle actin staining revealed very thin blood vessels walls in diclofenac treated mice. D and E, smooth muscle actin protein content in tumor homogenates (50 µg protein loaded per lane) of 4 untreated and 3 diclofenac treated mice measured as described in Materials and Methods, mean ± SE of arbitrary units/lane, * P≤0.01. F, Diclofenac reduces VEGF content of the tumor and the peritoneal cavity but not the serum. VEGF levels were measured as described in Materials and Methods in the tumor homogenates-pg/mg protein (*top*), in the peritoneal cavity-pg/mouse (*center*) and in the serum-pg/ml (*bottom*). The results are mean ± SE of 4 to 6 mice per group. *, ** and # significantly different from untreated, tumor bearing mice P≤0.02. Similar results were obtained in two independent experiments. G, Diclofenac inhibits vascular sprouting. The *ex vivo* inhibitory effect of diclofenac on sprouting was measured in rat aortic rings grown in the absence or the presence of 10 µM diclofenac for 5 days as described in Materials and Methods. The results are mean± SE of the sprout area of 5 rings in each group measured using the Image Pro program. * significantly different from untreated group P≤0.01 The photographs of representative rings from untreated (*left*) and diclofenac (*right*) treated groups are included. Similar results were obtained in 4 independent experiments.

Tumors from diclofenac treated animals were very pale (Fig 1A), suggesting that the treatment caused an antiangiogenic effect. Indeed, as shown in Fig 3A and B, staining for vascular endothelial marker von Willebrand Factor and counting of blood vessels revealed a 4 fold drop in the number of large peripheral vessels and a 2 fold drop in capillary density in tumors of diclofenac treated mice. Furthermore, smooth muscle actin staining (Fig 3C) revealed thin, straight vessels in treated tumors when compared to numerous, wide, tortuous blood vessels from tumors of untreated mice. Analysis of smooth muscle actin expression in tumor homogenates by western blot showed a significant decrease (2.5 fold) in diclofenac treated mice (Fig 3D and E).

VEGF has been shown to be the major proangiogenic factor in tumors [24]. Therefore, we measured tumor VEGF levels of diclofenac treated and untreated mice (Fig 3F). Treated mice tumors contained 3 times less VEGF when compared to tumors from untreated mice, suggesting that the decline in tumor vasculature results from a down regulation of VEGF (Fig 3F, *top*). Since tumor cells rapidly spread through the abdominal cavity, we next measured VEGF content in the peritoneal fluid. The flushable quantity of VEGF from the peritoneal cavity of tumor- bearing mice was 13 times higher than sham-operated healthy mice. This amount decreased by 2.8 fold after diclofenac treatment (Fig 3F, *center*).

These pronounced effects on VEGF content, both in peritoneal cavity and in tumor tissue, were not reflected in any changes in VEGF serum concentration (Fig 3F, *bottom*). Thus, serum VEGF concentrations were similar in serum of healthy-sham operated mice, in tumor- bearing mice and in tumor-bearing mice treated with diclofenac.

To determine whether diclofenac can directly inhibit angiogenesis, we measured sprouting angiogenesis of rat aortic rings *ex vivo* in response to drug treatment. As shown in Fig 3G, sprouting area was inhibited by 2.5 fold, when aortic rings were incubated with 10 µM of diclofenac (C max of diclofenac-treated patients), thus showing that diclofenac can directly inhibit blood vessel development.

### Diclofenac increases arginase activity in pancreatic tumors and in peritoneal macrophages, but not in bone marrow-CD 115 positive and CD 115 negative cells

**Figure 4:**
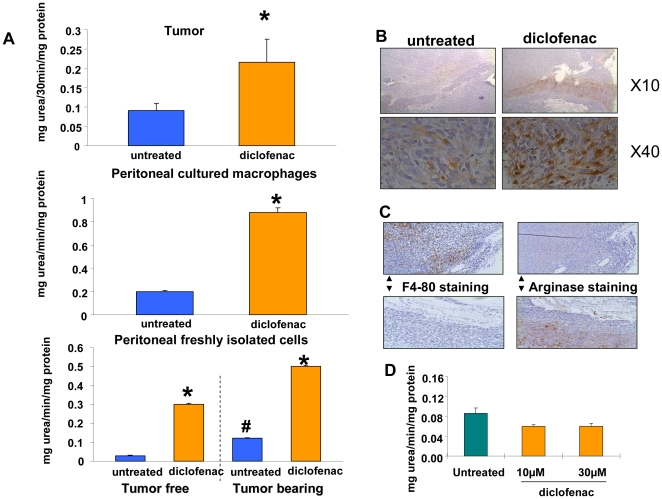
Diclofenac enhances arginase activity in tumors, peritoneal cultured macrophages and in freshly isolated peritoneal cells, but only when introduced *in vivo* and not *in vitro*. A–C, all mice except those indicated were inoculated with tumors on day 1 and killed on day 14. A, arginase activity was measured as described in Materials and Methods in tumor homogenate *(top)*, in macrophages isolated by peritoneal cavity flush and cultured for 20 hrs (*center)*, and in freshly isolated peritoneal cells (*bottom)*. Mice were treated with diclofenac 30 mg/kg for 11 days (*top and center*) or for 2 days before the end of the experiment (*bottom*). The results are mean± SE of arginase activity in tumor homogenates of 6–7 mice in each group (*top*) or in Triton X-100 dissolved cells obtained from 3–7 mice through peritoneal flush and measured in quadruplicates (*center and bottom*). Each experiment was repeated at least 3 times and the results of the representative experiment are shown. * significantly different from untreated group P≤0.01. # significantly different from untreated group of tumor- free- mice. B, arginase-1 staining of tumors. Fixated tumors were stained for arginase 1 as described in Materials and Methods. Photographs of sections were taken under X 10 *(top)* or X 40 (*bottom*) magnification. C, The identification of two separate populations of cells in tumor (diclofenac-treated) stroma: F4-80 positive and arginase positive cells. The serial sections were stained for F4/80 *(left*) and for arginase 1, *(right)*. The same areas were identified and compared for the presence of both markers. Photographs of sections were taken under X 20 magnification. One experiment, representative of 2. D, Diclofenac does not induce arginase activity when incubated with macrophages in cell culture. Peritoneal macrophages were isolated from tumor-free mice as described in Materials and Methods and incubated for 48 hours with 10 or 30 µM diclofenac followed by arginase measurement. Mean ±SE of arginase activity of 3 wells in each group. One experiment, representative of 3.

One of the results of COX-2 overexpression by tumor cells is a large production of PGE2 which leads to an impaired T-cell response [14] [25]. PGE2 induces arginase 1 activity and arginine uptake in myeloid derived suppressor cells (MDSCs), thus causing arginine depletion in the tumor surrounding. The relative lack of arginine causes a defect in the CD3ζ expression of the tumor-infiltrating T cells. Since COX-2 inhibitors were shown to partially stop tumor growth through arginase inhibition in MDSC [14], [25] we measured arginase activity in pancreatic tumor homogenates from non-treated and diclofenac treated mice (Fig 4A, *top*).

To our surprise arginase activity was not inhibited by diclofenac treatment, but rather was significantly activated by 2.4 fold.

Immuno-histological staining showed arginase 1 positive cells at the periphery of tumors in both untreated and diclofenac treated mice (Fig 4B). The number of arginase 1 expressing cells and their staining intensity seemed to increase under diclofenac treatment. A significant, 1.8 fold increase in arginase 1 protein content in tumors of diclofenac treated mice was also measured by western blot analysis of tumor homogenates (Fig S2). Using an antibody against the macrophage marker F4/80 (Fig 4C) we could not detect any overlap with arginase 1 staining.

In order to further characterize the arginase 1 expressing cells in PANC02 tumors, we inoculated PANC02 cells into transgenic mice where a GFP reporter is driven by the CX3CR1 promoter. Activity of this chemokine receptor promoter is restricted to mononuclear myeloid cells, including all circulating CD116^+^ monocytes, and is essentially absent from cells of the lymphoid lineage. We isolated GFP positive cells from tumors and found that these cells express high levels of arginase 1 (Fig S3).

Thus, the increased activity of arginase found in tumors from diclofenac treated mice is due at least in part to the activation of arginase 1 in monocyte derived CX3CR1^+^ cells. Arginase activity was absent in cultured PANC02 (results not shown) and arginase 1 protein was never detected in tumor cells *in vivo* by immunostaining.

The striking effect of diclofenac treatment on arginase activity in pancreatic tumors led us to examine arginase activity in peritoneal macrophages. Peritoneal macrophages can be involved in tumor surveillance [26] and show an enhanced arginase expression in tumor-bearing mice [27]. Macrophages from peritoneal lavage were isolated by preferential attachment to culture dishes and cultured overnight. Immunostaining showed that they were both F4/80 positive and arginase 1 positive (results not shown). Arginase activity was upregulated (4.8 fold) in macrophages derived from tumor- bearing mice treated with diclofenac for 11 days, when compared to untreated mice, (Fig 4A *center*).

Since macrophage arginase expression can change in response to culture dish adhesion [28] we also measured arginase activity in non-cultured freshly isolated cells from the mouse peritoneal cavity, thereby compromising on macrophages purity, (Fig 4A *bottom)*.

Diclofenac effectively stimulated arginase activity in non-cultured cells derived from both tumor-free and tumor-bearing mice treated with diclofenac for 2 days only by 7 and 3 fold, respectively. Tumor presence enhanced arginase activity by 4 fold increasing the activity from 0.03 (cells from non-tumor bearing mice) to 0.12 mg urea/min/mg protein (cells from tumor -bearing mice) which is in line with previous reported results [27].

We also counted the number of peritoneal cells extracted from each mouse and found that a 2 day diclofenac treatment yielded a 3 fold increase in cells from tumor-free mice. The mean number of cells±SE was 0.6×10^6^±0.06×10^6^ extracted from untreated mice and 1.5×10^6^±0.4×10^6^ from diclofenac treated mice (p≤0.02, 7 mice in each group).

This observation shows that after a short diclofenac treatment, the total arginase activity in peritoneal cells (mostly macrophages) is extremely high, both because of the large increase in a specific activity of this enzyme and because of the increase in the number of activated cells.

The activation of the arginase activity by diclofenac could not be demonstrated *in vitro*. Incubation of peritoneal macrophages for 48 hours (Fig 4D) did not increase arginase activity, but rather yielded a certain decrease in the specific activity of this enzyme. The same results were obtained when macrophages were incubated for 96 hours with diclofenac (results not shown). Thus, similarly to the effect of diclofenac on apoptosis of tumor cells, mediators present *in vivo* and absent in cultured macrophages were required in order to achieve the induction of arginase activity by this drug.

We also investigated whether arginase activation by diclofenac can be detected in bone marrow macrophage precursors. We isolated mononuclear cells from tibias and femurs of tumor-bearing mice untreated and treated for 11 days with diclofenac. We found very low arginase activity in both CD 115^+^ and CD 115^−^ cells. Thus, arginase activation by diclofenac happens either in differentiated macrophages or the mediators needed for promoting diclofenac- induced- activation of arginase do not reach the bone marrow compartment.

### Diclofenac decreases NO level in peritoneal cavity and in serum

**Figure 5:**
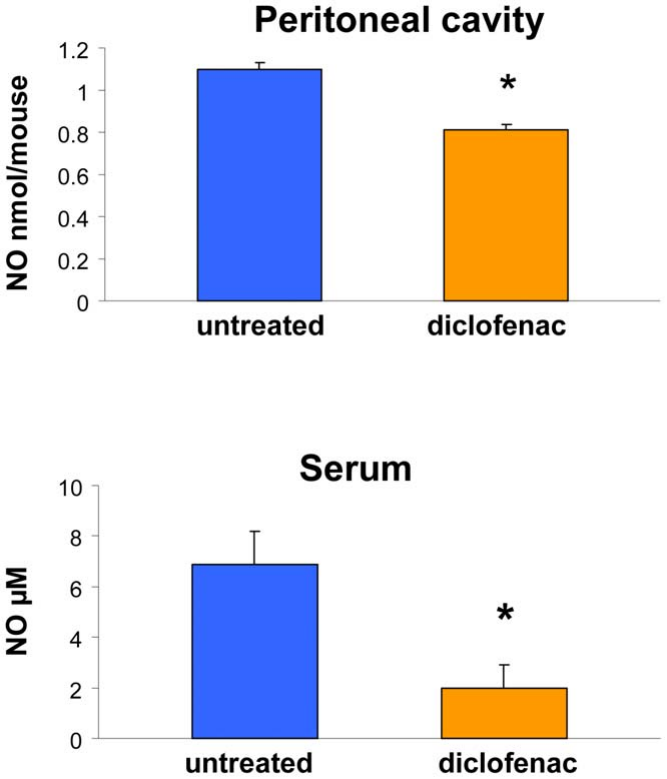
Diclofenac decreases NO content in peritoneal cavity and in serum. Tumor bearing mice, untreated and treated with diclofenac 30 mg/kg b.w. for 11 days were sacrificed on day 14 after tumor inoculation. The peritoneal cavity was flushed and blood was collected by heart puncture. The results are mean± SE of NO content in peritoneal cavity (*top*) and serum (*bottom*) of 7–9 mice in each group. * significantly different from the untreated group P≤0.01.

We next investigated whether the pronounced activation of arginase in both tumor tissue and in peritoneal macrophages influenced NO production. Arginase and nitric oxide synthase (NOS) compete for arginine, which serves as substrate for both these enzymes. The activation of arginase can therefore lead to arginine depletion [29] yielding a decrease in NOS activity. Indeed, our measurements of the NO content in the peritoneal cavity (Fig 5*top*), and in the serum (Fig 5*bottom* ), showed a significant decrease in NO in both these compartments. The NO content in the peritoneal cavity was 1.3 fold lower in tumor-bearing mice treated for 11 days with diclofenac, as compared with tumor-bearing untreated mice. The effect on serum NO concentration was even more pronounced and amounted to 3.5 fold drop in diclofenac treated animals.

In order to study the reason for this very high drop in NO serum concentration we isolated white blood cells (WBC) from untreated and diclofenac treated mice. We found that diclofenac treated mice contained 4 fold higher quantity of WBC per milliliter of blood. The mean number of cells ±SE was 0.26×10^6^±0.03×10^6^ isolated from untreated mice and 1.2×10^6^±0.09×10^6^ from diclofenac treated mice (P≤0.001, 3 mice in each group).

The arginase protein level per equal number of cells was increased 2 fold Fig S4. Thus, arginase activity in diclofenac treated mice blood is about 8 fold higher as compared to untreated mice, and may explain a pronounced decrease in NO serum concentration.

## Discussion

In this study we have examined the antitumor potential of diclofenac in an orthotopic model of mouse pancreatic cancer. PANC02 cells injected into the tail of the pancreas rapidly multiplied, formed large local tumors and repeatedly metastasized primarily to the cut in the abdomen performed for inoculation (Fig 1). The tendency of tumor cells to implant into a wound is a concern in cancer surgery [30] and has been studied in animal models [31]. The pro-tumor factors secreted by wounds may be responsible for this phenomenon.

Diclofenac treatment caused a significant decrease in the tumor weight at the primary site of injection. This decrease was due to enhanced apoptosis of cancer cells (Fig 2). The anti-tumoral capacity of diclofenac is generally attributed to its direct effect on tumor cell viability and cell cycle [32], [33]. However this effect is obtained only using very high concentrations of drug *in vitro* (up to 800 µM). This may not reflect the *in vivo* situation except for topical administration of the drug [32].

In our model, the effect on PANC02 growth rate could not be reproduced *in vitro*, when cells were incubated either in the presence of 10 µM diclofenac, the higher range of C max values of the drug reported in human [34] and mice [35] studies, or in the presence of 50 µM of the drug. This prompted us to test the activity of diclofenac on other tumor growth promoting components.

Antiangiogenic activity of diclofenac was suggested in a model of subcutaneous implanted colon-26 adenocarcinoma cells, where the drug was topically applicated [32]. Our study further demonstrates the antiangiogenic potential of diclofenac in intra abdominal tumors. We found, that after 11-day treatment, diclofenac caused a significant decrease in blood content of tumors as illustrated by their pale appearance (Fig 1), decrease in blood vessels count and smooth muscle actin content (Fig 3B and Fig 3C, D and E).

VEGF has been shown to be the main angiogenic factor in tumor vascularization. Therefore we measured VEGF protein levels both in the tumor and in the peritoneum and found a pronounced decrease in diclofenac treated animals (Fig 3F). The decrease in VEGF content in tumors of diclofenac treated mice may be due to a decrease in VEGF expression in tumor cells, in stroma cells, or in both types of cells. The decrease in the peritoneal cavity can be due to the change in VEGF content of tumor or to the direct effect of diclofenac on peritoneal mesothelial cell lining. Additional studies are required to resolve this issue. The effect of diclofenac on VEGF production could not be recapitulated *in vitro* when cultured PANC02 or peritoneal macrophages were incubated with diclofenac (Fig S5). Previously, diclofenac has been reported to decrease mRNA-VEGF levels via COX inhibition in cultured esophageal cancer cell lines [36]. In our *in vivo* model it is thus likely that the direct effect of diclofenac on VEGF production leads to an antiangiogenic effect which in turn affects apoptosis of cancer cells in the growing tumor.

We have also tested the possibility that diclofenac may directly affect vascular cells. While diclofenac was unable to induce pro-apoptotic and anti-VEGF effect on cultured PANC02, its antiangiogenic effect could be demonstrated *ex vivo* (Fig 3G). When 10 µM diclofenac was incubated together with rat aortic rings, it yielded a pronounced inhibition of sprouting. This suggests that diclofenac can directly inhibit the development of endothelial/smooth muscle cells.

Interestingly, we have found that peritoneal concentration of VEGF is significantly higher than that of the serum, both in tumor-free and in tumor-bearing mice. Assuming that the volume of the peritoneal fluid is about 100 µl [37], our results demonstrate that the VEGF concentration amounts to about 340 pg/ml in sham-operated-tumor-free mice and to above 4000 pg/ml in tumor-bearing mice. The high concentration of VEGF in the peritoneal cavity does not equilibrate with the blood compartment and is 4 and 55 fold higher (as compared with serum values) in sham-operated and tumor-bearing mice, respectively. In healthy pigs [38] it was also shown that the intra peritoneal VEGF does not equilibrate with the serum. Our results, as well as study [39], challenge the notion that systemic VEGF levels can predict tumor load. Furthermore it may be interesting to explore the possibility of delivering antiangiogenic antibodies for intra abdominal tumors directly into the peritoneal cavity and not systemically.

Unlike the clear antineoplastic effect of diclofenac through inhibition of angiogenesis, the striking activation of arginase activity (Fig 4), both in tumor stroma and in peritoneal macrophages is difficult to assess in context of pro- or anti-cancer activity. One study noted that high arginase activity found in myeloid derived suppressive cells (MDSC), was associated with immunosuppressive activity and tumor-promotion [25]. The authors proposed that high COX-2 activity of tumor cells releases PGE2, which activates arginase in MDSC and leads to extracellular arginine depletion and subsequent T-cell dysfunction. Along with these results, two additional studies [14], [15] have shown that treatment of tumor bearing mice with COX-2 specific inhibitors completely blocked the induction of arginase activity, reduced MDSC accumulation and decreased tumor growth. However, there is evidence that tumor cells are particularly sensitive to arginine depletion [40] and that enhanced arginase activity can restrict tumor growth [18] and [16]. Furthermore it has been shown that dietary arginine restriction caused a significant reduction in both tumor incidence and tumor multiplicity in a mouse papilloma cancer model [41].

To our knowledge we are the first to show that diclofenac causes pronounced arginase activation in tumor stroma CX3CR1 ^+^ cells, in peritoneal macrophages and in WBC. This activation correlates with a significant inhibition of tumor growth. Further research is needed in order to determine if this correlation is causative or coincidental.

Our study supports the development of a new therapeutic avenue through the use of pegylated recombinant human arginase [18], [19].

## Supporting Information

Figure S1 Diclofenac does not affect proliferation. Mice were inoculated with PANC02 cells and treated with diclofenac as described in Figure 1. Ki 67 and phosho-histone H3 staining of fixed tumors was performed as described in Materials and Methods. Ki 67 was quantified (% of Ki positive nuclei) with aid of image analysis system (Ariol SL-50). The number of phospho-histone H3 positive cells/area was quantified by counting 10 different areas under X 20 magnification. Evaluations were performed in tumors of 4 untreated and 4 diclofenac treated mice.
(4.54 MB TIF)

Figure S2 Diclofenac increases arginase 1 protein content in tumor homogenates. Mice were inoculated with PANC02 cells and treated with diclofenac as described in Figure 1. Tumor homogenates from 5 untreated and 6 diclofenac treated mice were prepared and arginase 1 protein content was analyzed using Western blott as described in Materials and Methods. Mean±SE of arbitrary units/lane. 50 µg protein of tumor homogenate was loaded per lane. * significantly different from the untreated group P≤0.001.
(0.17 MB TIF)

Figure S3 Arginase 1 positive cells are of mononuclear myeloid cells origin (CX3CR1 positive). PANC02 cells were inoculated into CX3CR1^GFP/+^ mice. After 14 days tumors were extracted, digested and GFP positive and negative cells were isolated by high speed cell sorting using FACS, as described in Materials and Methods. 400 000 cells GFP positive and negative cells were counted, lysed and analyzed for arginase 1 protein content as described in Materials and Methods.
(0.09 MB TIF)

Figure S4 Diclofenac increases WBC arginase 1 content. CB6F1 mice (tumor-free) were treated for 6 days with 30 mg/kg b.w. diclofenac. WBC were isolated from 1 ml of blood using Ficoll density gradient as described in Materials and Methods and counted. 200 000 cells were lysed and analyzed for arginase 1 protein content. Mean± SE of arbitrary units/lane of 3 untreated and 3 diclofenac treated mice. *significantly different from the untreated group P≤0.05.
(0.19 MB TIF)

Figure S5 Diclofenac does not affect VEGF production in PANC02 or macrophages in vitro. A, PANC02 (3000 cells/well) were seeded in 96−NUNC wells. The day after seeding 10 or 50 µM diclofenac was added and cells were incubated for additional 4 days. B, Peritoneal macrophages were isolated from tumor−free mice as described in Materials and Methods and incubated for 48 hours with 10 or 50 µM diclofenac. At the end of the incubation cells were washed, lysed and measured for VEGF content as described in Materials and Methods. Mean± SE of pgVEGF/mg protein in 6 wells of untreated and diclofenac incubated cells.
(0.17 MB TIF)
