# Supplementary figures and images for: Diclofenac Inhibits Tumor Growth in a Murine Model of Pancreatic Cancer by Modulation of VEGF Levels and Arginase Activity
Source: PLoS One. 2010 Sep 15;5(9):e12715. doi: 10.1371/journal.pone.0012715 (PMC2939880; doi:10.1371/journal.pone.0012715)

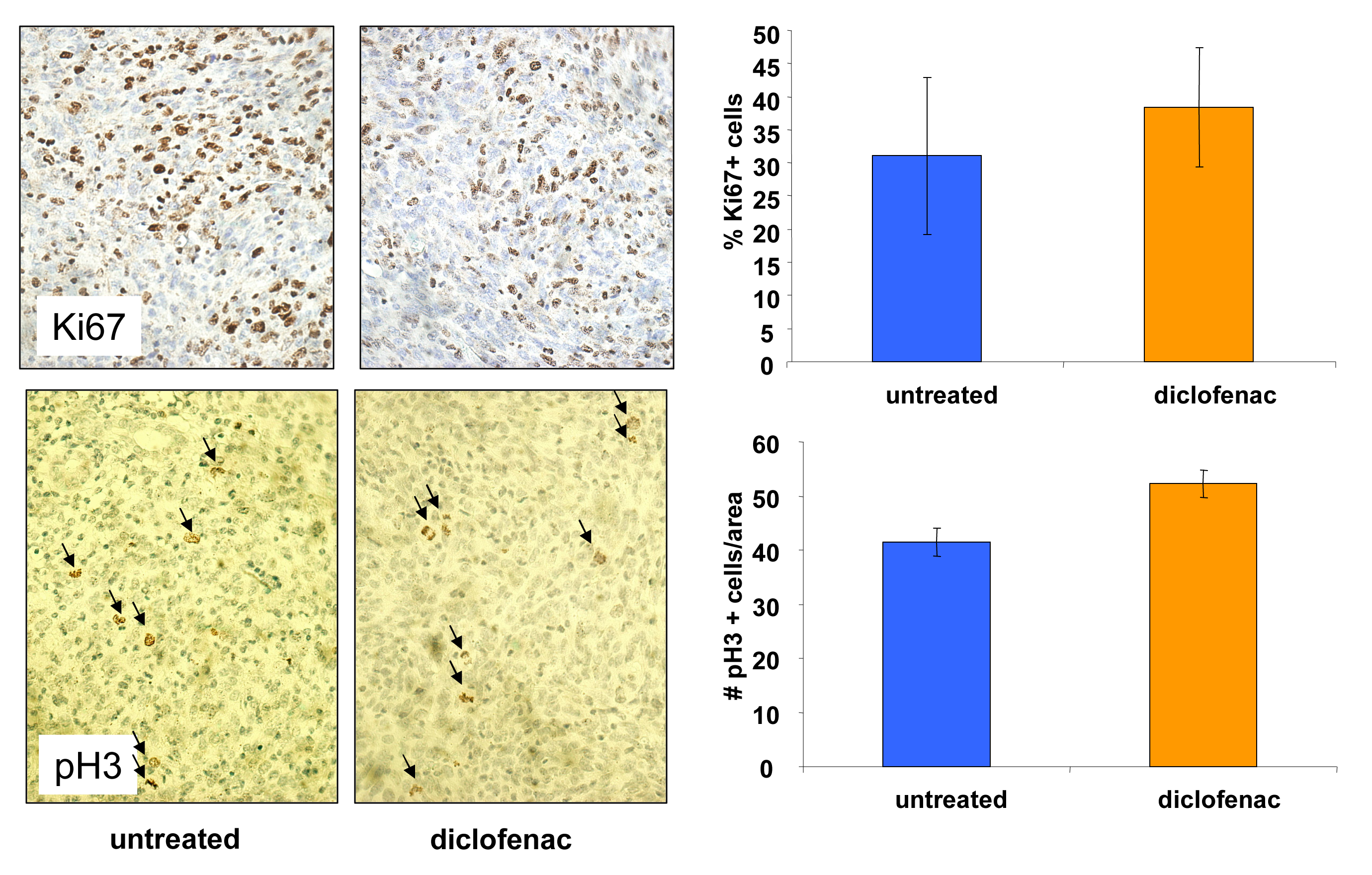

Supplement: Figure S1 — Diclofenac does not affect proliferation. Mice were inoculated with PANC02 cells and treated with diclofenac as described in Figure 1. Ki 67 and phosho-histone H3 staining of fixed tumors was performed as described in Materials and Methods. Ki 67 was quantified (% of Ki positive nuclei) with aid of image analysis system (Ariol SL-50). The number of phospho-histone H3 positive cells/area was quantified by counting 10 different areas under X 20 magnification. Evaluations were performed in tumors of 4 untreated and 4 diclofenac treated mice. (4.54 MB TIF) [file pone.0012715.s001.tif]

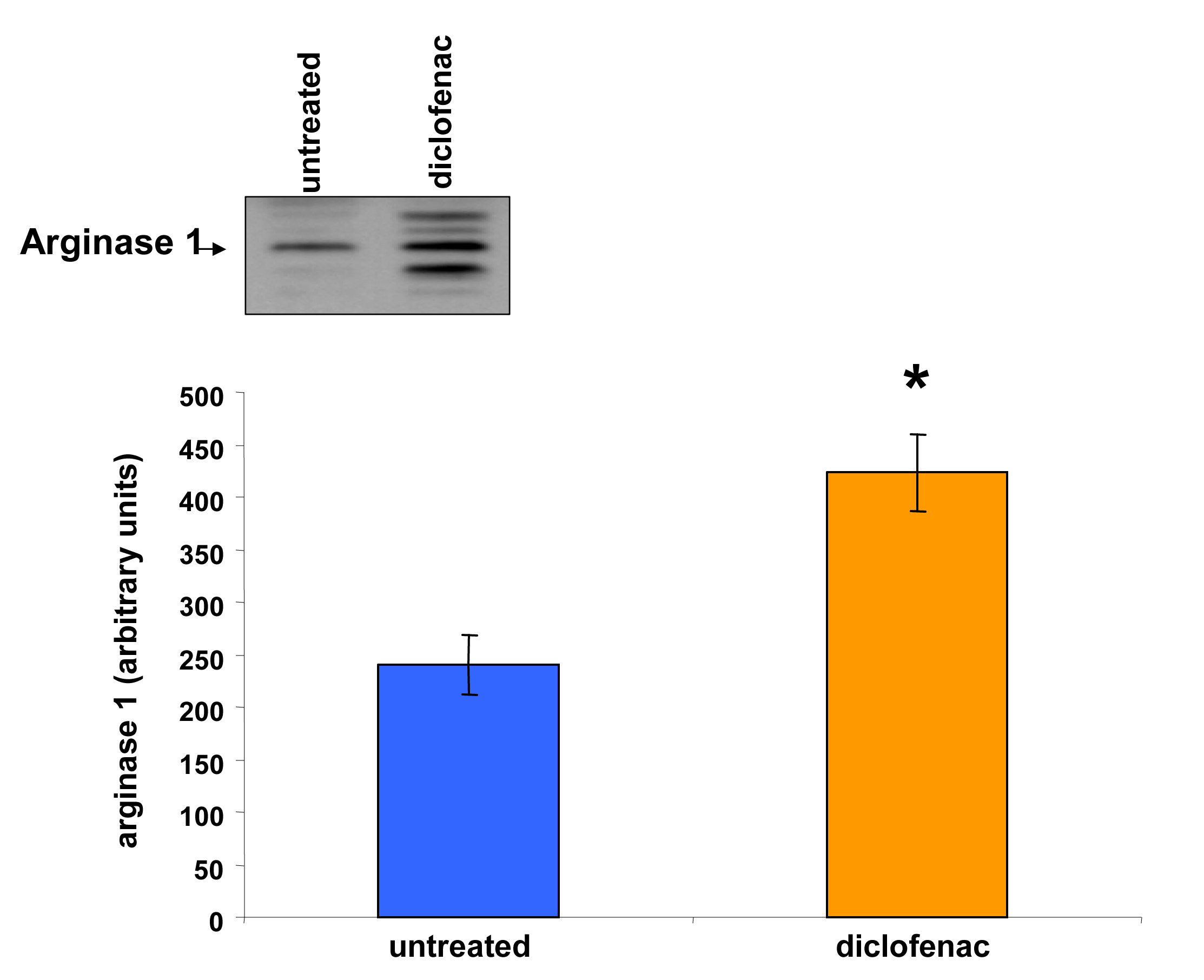

Supplement: Figure S2 — Diclofenac increases arginase 1 protein content in tumor homogenates. Mice were inoculated with PANC02 cells and treated with diclofenac as described in Figure 1. Tumor homogenates from 5 untreated and 6 diclofenac treated mice were prepared and arginase 1 protein content was analyzed using Western blott as described in Materials and Methods. Mean±SE of arbitrary units/lane. 50 µg protein of tumor homogenate was loaded per lane. * significantly different from the untreated group P≤0.001. (0.17 MB TIF) [file pone.0012715.s002.tif]

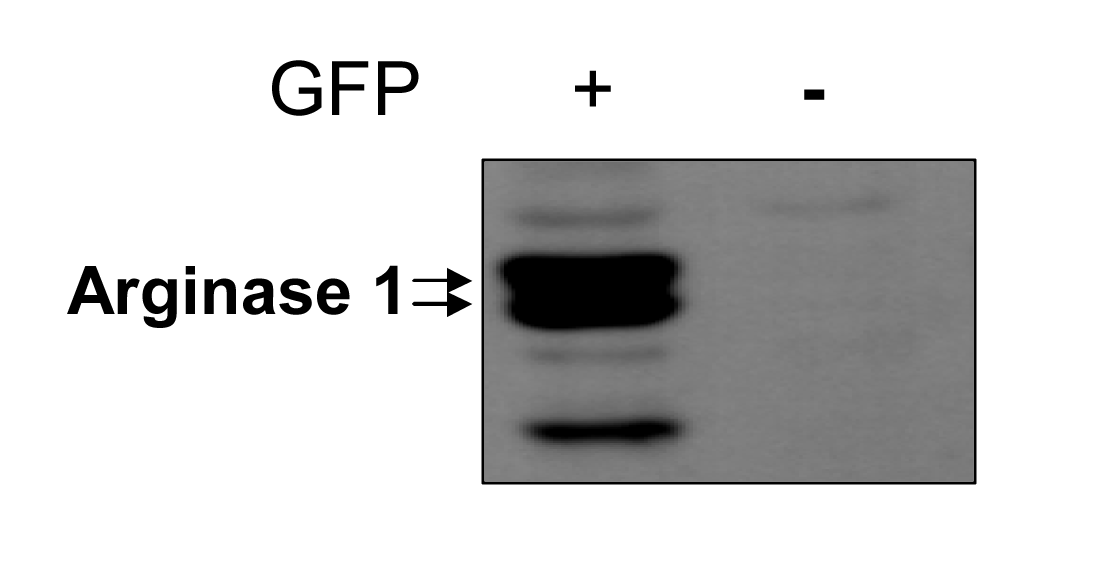

Supplement: Figure S3 — Arginase 1 positive cells are of mononuclear myeloid cells origin (CX3CR1 positive). PANC02 cells were inoculated into CX3CR1GFP/+ mice. After 14 days tumors were extracted, digested and GFP positive and negative cells were isolated by high speed cell sorting using FACS, as described in Materials and Methods. 400 000 cells GFP positive and negative cells were counted, lysed and analyzed for arginase 1 protein content as described in Materials and Methods. (0.09 MB TIF) [file pone.0012715.s003.tif]

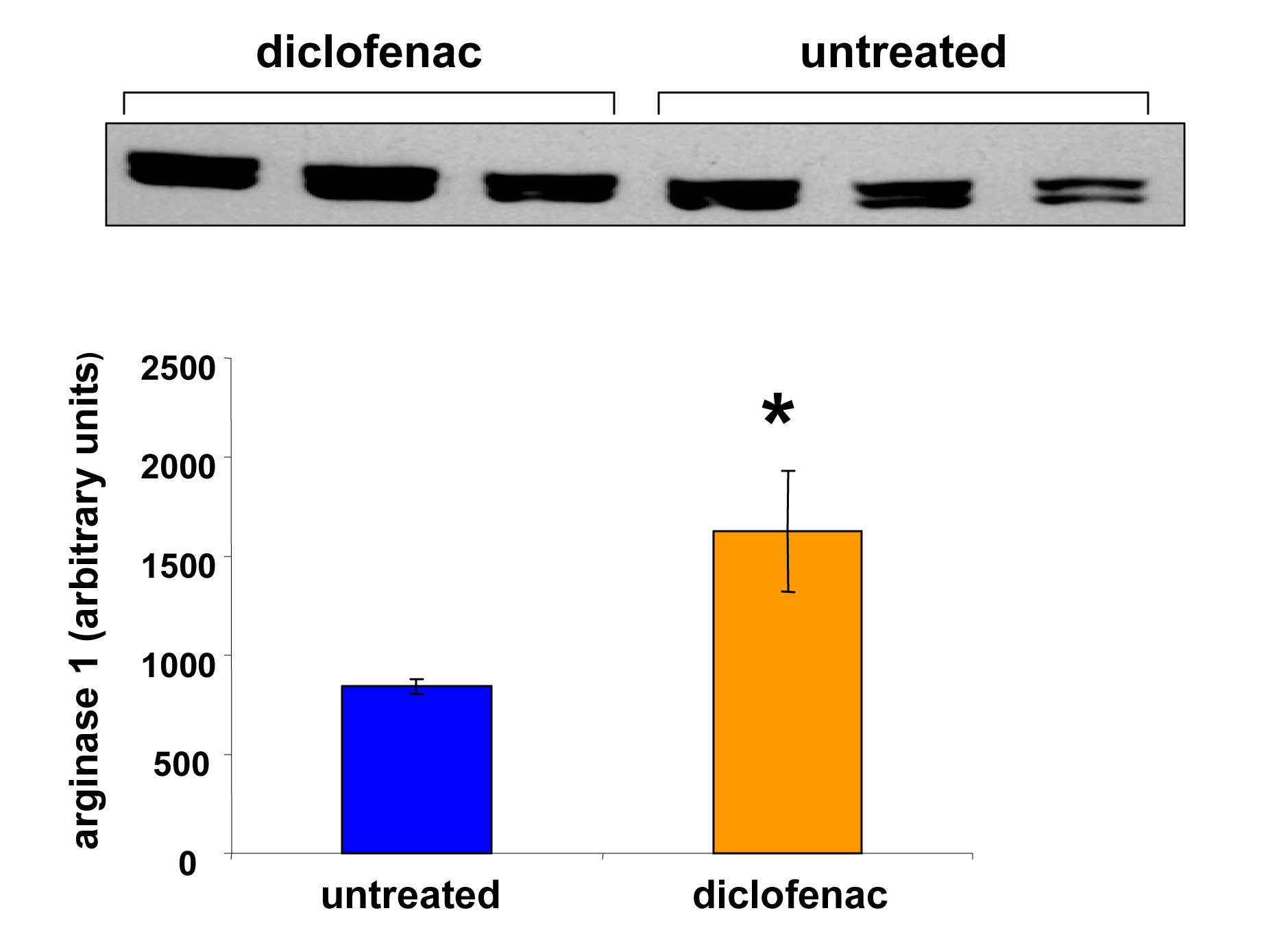

Supplement: Figure S4 — Diclofenac increases WBC arginase 1 content. CB6F1 mice (tumor-free) were treated for 6 days with 30 mg/kg b.w. diclofenac. WBC were isolated from 1 ml of blood using Ficoll density gradient as described in Materials and Methods and counted. 200 000 cells were lysed and analyzed for arginase 1 protein content. Mean± SE of arbitrary units/lane of 3 untreated and 3 diclofenac treated mice. *significantly different from the untreated group P≤0.05. (0.19 MB TIF) [file pone.0012715.s004.tif]

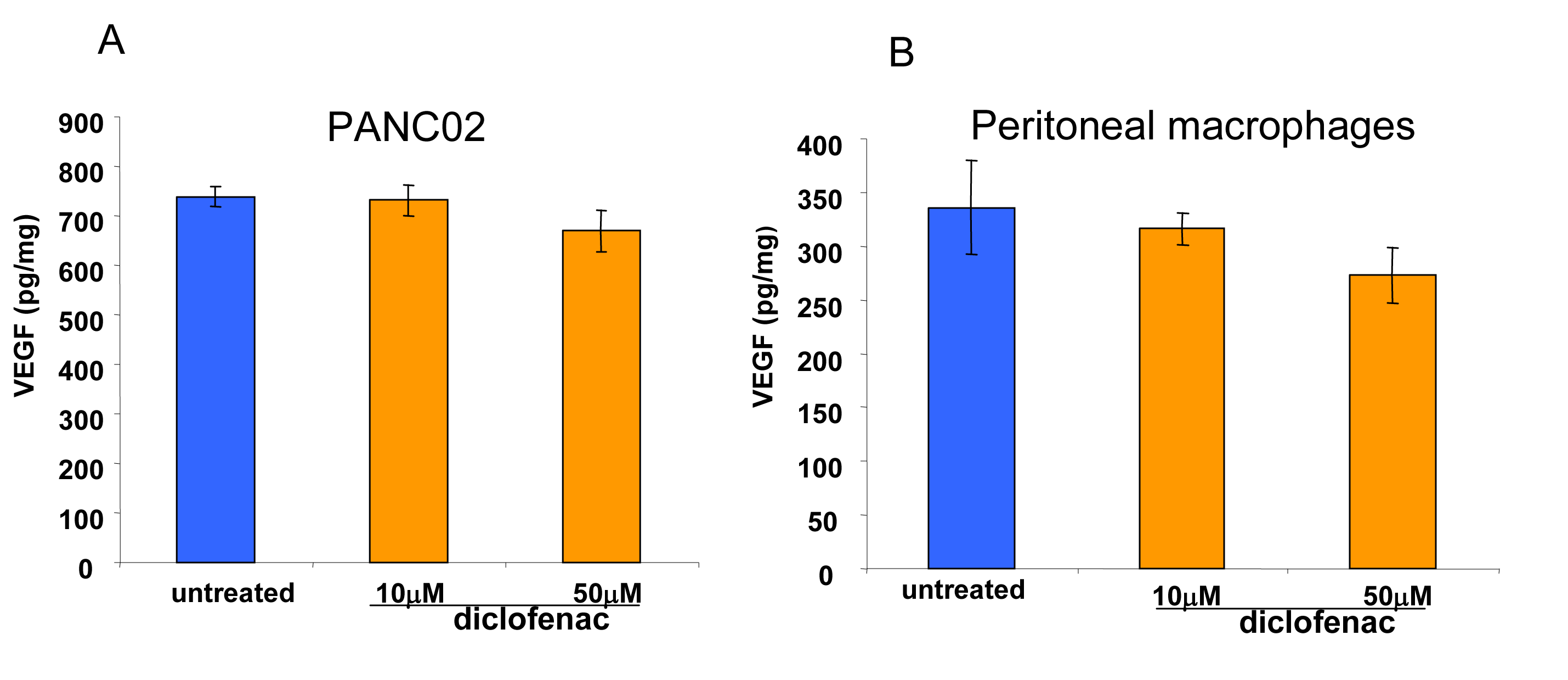

Supplement: Figure S5 — Diclofenac does not affect VEGF production in PANC02 or macrophages in vitro. A, PANC02 (3000 cells/well) were seeded in 96−NUNC wells. The day after seeding 10 or 50 µM diclofenac was added and cells were incubated for additional 4 days. B, Peritoneal macrophages were isolated from tumor−free mice as described in Materials and Methods and incubated for 48 hours with 10 or 50 µM diclofenac. At the end of the incubation cells were washed, lysed and measured for VEGF content as described in Materials and Methods. Mean± SE of pgVEGF/mg protein in 6 wells of untreated and diclofenac incubated cells. (0.17 MB TIF) [file pone.0012715.s005.tif]
